# Diagnosis and Management of Rare Siliconomas in an HIV Patient

**DOI:** 10.7759/cureus.14645

**Published:** 2021-04-23

**Authors:** Rohit Munagala, Pranjal Mishra, Akash Chakravartty, Arjun N Bhatt, Jayanth Keshavamurthy

**Affiliations:** 1 Radiology, Augusta University Medical College of Georgia, Augusta, USA

**Keywords:** siliconoma, silicone injection, hiv

## Abstract

Siliconomas are rare conditions stemming from uses of silicone injections for soft tissue augmentation, most commonly in the breast and buttocks areas. Siliconomas are known to present with suspicious morphology that mimics cases of embolism or systemic metastasis as the silicone travels through blood and lymphatics. We present the case of a 45-year-old HIV-positive male who presented with siliconomas in the breast region, chest heaviness, shortness of breath, dyspnea, and a physical exam showing gynecomastia. The patient denied any surgeries or injections around his chest. Further imaging showed abnormal fat deposition in the chest and possible metastatic lymphadenopathy to axillary, supraclavicular, and mammillary lymph nodes. Although the complications arising from silicone injections are well documented, the pathogenesis remains unknown, leaving a narrow range of therapeutic options. Despite these shortcomings, diagnostic imaging tools have shown to be vital in the diagnosis and localization of suspected siliconomas.

## Introduction

Traditionally, silicone is considered to be biologically inert and essentially harmless; however, more recent studies indicate silicone products are potentially bioactive agents that may induce a variety of host reactions [1]. There is no consensus on the mechanism of action, biological activity, or the long-term significance. Silicone injections were once commonly used for soft tissue augmentation, but this is no longer approved for that purpose in the United States by licensed physicians. Current FDA regulations only indicate silicone injections temporarily for retinal attachments, with removal of the substance after attachment [1]. However, these injections are still frequently performed by non-physicians in regions of the United States without proper medical supervision [2].

The majority of those who received silicone injection began to experience side effects of silicone 5-20 years after the initial treatment, but complications have been reported as long as 36 years following treatment [2]. A common side effect is the benign spread of the silicone after seeding nearby lymphatics, specifically in breast tissue [2]. Positron emission tomography (PET) scans have been shown useful as silicone granulomas manifest as multiple masses with increased Fluorodeoxyglucose (FDG) uptake [2]. Biopsies of these tissues present with numerous macrophages, multinucleated giant cells, and large clear vacuoles disseminated in breast lobules and stroma [1]. Although no treatment methods are officially agreed upon, current methods include surgical resection with fat grafting to replace resected tissues, and post-surgical corticosteroid injections to contain hypertrophic scarring [3]. With the increased vascularity of the buttocks area, silicone injections in perivascular tissue of the buttocks have been shown to embolize to pulmonary capillaries resulting in alveolar hemorrhage and inflammation, commonly referred to as Silicone Embolization Syndrome [4].

Here, we present a case of an African American male with a history of HIV who was initially diagnosed with HIV lipodystrophy, but radiographic studies of the breast ultimately showed the presence of siliconomas.

## Case presentation

**Figure 1:**
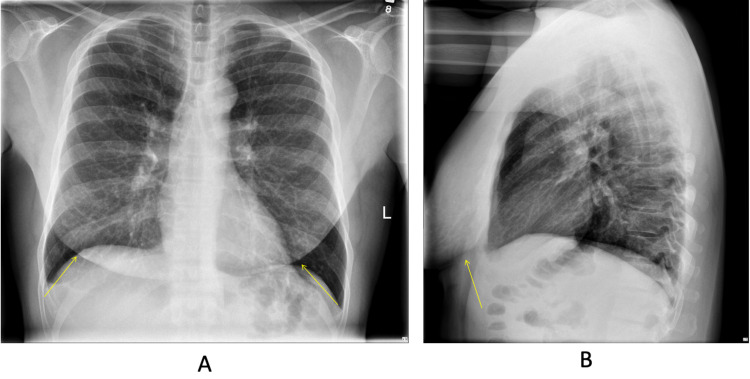
Frontal (A) and lateral (B) chest radiographs show bilateral symmetric soft tissue densities.

**Figure 2:**
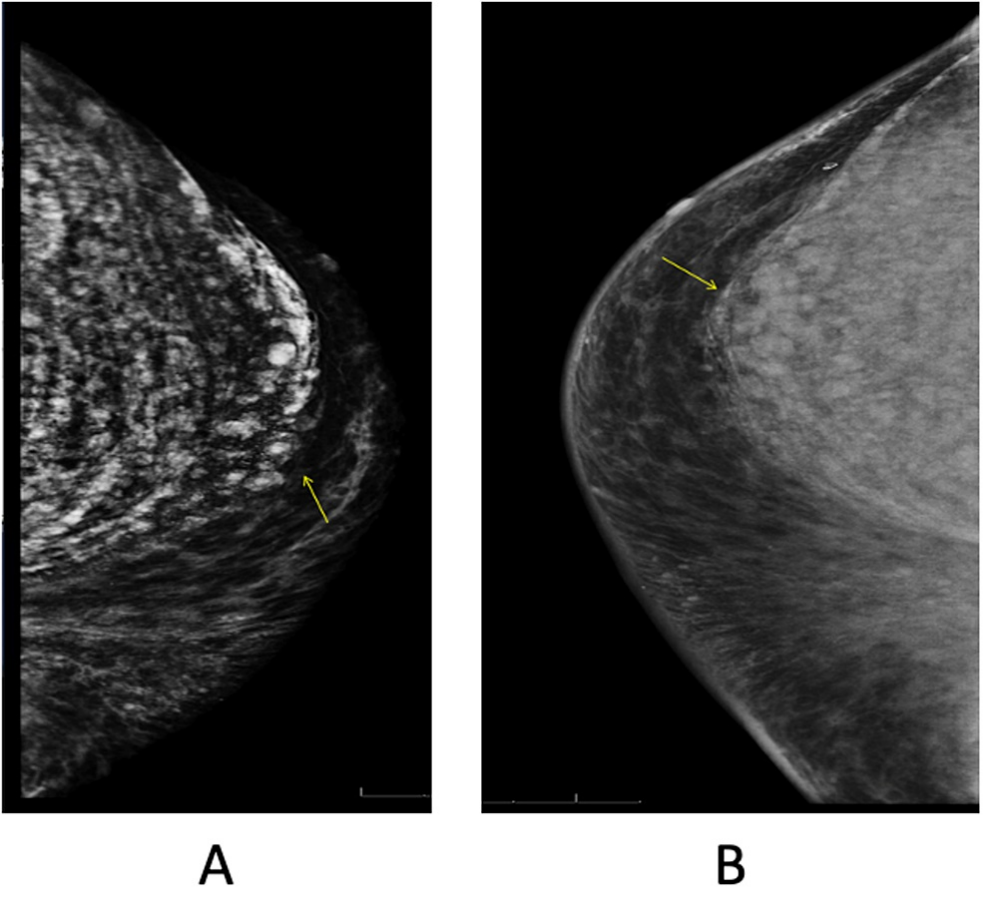
Left (A) and right (B) mammograms show calcifications within the pectoralis muscles.

A 45-year-old African American male presented to primary care for chest heaviness, shortness of breath, and dyspnea. Past medical history was significant for the history of HIV infection and the current medical regimen included darunavir, etravirine, lamivudine, zidovudine, and emtricitabine. On physical examination, the patient was a well-appearing male with normal vital signs but was found to have an abnormally large amount of fat deposition around the chest, initially thought to be chest lipodystrophy related to HIV medications. He denied any prior surgery, injections, or augmentations in his chest region. Initial chest x-ray showed large amounts of soft tissue density overlying the anterior chest (Figures 1A, 1B). CT thorax with contrast revealed a pleural-based nodule in the right lung that was not previously present, increased anterior thoracic and abdominal wall soft tissue edema, and ground-glass opacities in the superior segment of the left lower lobe. Mammograms showed extensive calcification within the pectoral muscles bilaterally, without breast masses or nodules (Figures 2A, 2B). CD4 count was 305 cells/mm^3^ and most recent viral load showed <40 copies/mL indicating suppressed viremia. His endocrine and hematology panel were unremarkable. The chemistry panel revealed serum protein level of 8.8, albumin 4.5, and aspartate aminotransferase (AST) of 40.

**Figure 3:**
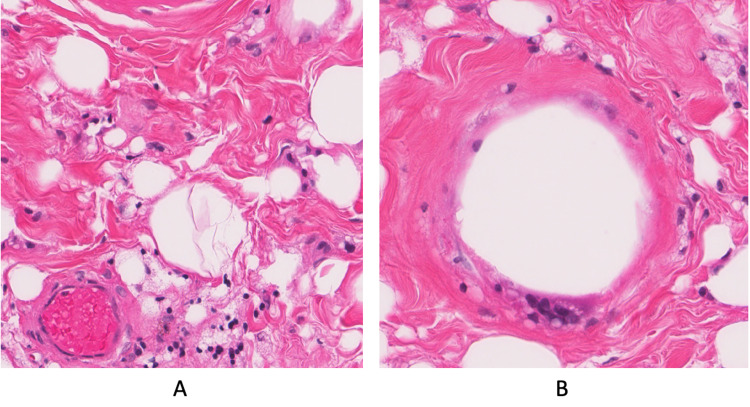
Fine vacuoles were visualized from locations where silicone had previously dissolved via hematoxylin-eosin stain.

The patient returned one month later with worsening shortness of breath and chest heaviness. On physical exam, the patient was found to have interval enlargement of both breasts as well as a midline anterior chest wall mass. The patient underwent bilateral mastectomy and excision of the mid-anterior chest mass, which measured 15 x 12 cm. While excess breast tissue was being removed, cysts were discovered that were noted to be of the same consistency as siliconomas. The dissection was difficult to continue due to silicone-associated fibrosis and scaring. A total of 314 grams of tissue was removed from the right breast and mid-chest mass, while 185 grams of tissue was removed from the left breast. Biopsy of the chest wall and breast tissue showed fibroadipose tissue with extensive fat necrosis and lymphocytes (Figures 3A, 3B). Additionally, giant cell reaction, foamy microphages, and fibrotic, partially calcified microcysts were visualized. Fine vacuoles were noted and determined to be from locations where silicone had previously dissolved. Biopsies were negative for malignancy. Pathology was determined to be consistent with the clinical picture of siliconoma. At post-surgical follow-up, the patient stated he no longer felt short of breath or heavy. While initially not mentioned by him, the patient admitted to prior silicone injection use for breast augmentation upon further questioning.

Patient consent was obtained through a legal, written consent form before using patient information to construct a case report. The patient was compliant with the use of his case for research purposes.

## Discussion

Siliconomas can form after the rupture of a silicone-based implant. The silicone released from the implant can interact with the surrounding tissue and induce a granulomatous foreign body reaction [4]. The mass that consequently forms can often mimic cancer [4].

Because siliconomas have no defining trait in clinical presentation and can appear years or even decades after an implant, a patient history of silicone-based implants will be critical in guiding clinicians to include this diagnosis on their differential. However, many patients will not be willing to admit to a history of implants, which can complicate diagnosis. Siliconomas are most likely to form where silicone-based implants are placed, and the breasts are one of the most common locations for implants. Accordingly, the formation of an unusual breast mass inconsistent with clinical history may warrant the inclusion of siliconoma on the differential diagnosis. 

As siliconomas can often mimic breast cancer, radiographic studies will often be required to exclude malignancy. MRI is the radiographic tool of choice, and any patient with a history of silicone implants presenting with a breast or axillary mass should undergo an MRI. The classic MRI findings of siliconomas include free silicone particles outside the prosthetic shell and inside the surrounding tissue [2]. Such a radiological finding can often make the diagnosis of siliconoma and allow for the exclusion of malignancy. On FDG-PET, the inflammatory cells of a siliconoma exhibit increased FDG uptake and may thus mimic malignancy. The inflammatory cells of a siliconoma undergo heavy glycolysis and exhibit increased FDG uptake, which can lead to a false-positive result. As such, caution must be exercised in patients who may potentially have a siliconoma; MRI is the radiographic tool of choice for these patients. 

The diagnostic gold standard for siliconoma is tissue biopsy with histological analysis. Injected silicone microdroplets stimulate collagen formation and can induce fibrosis and scarring. Associated histological findings include calcified microcysts, giant cell granulomatous reactions, and fibrous tissue with fat necrosis and lymphocytes. 

Treatment of siliconoma can vary. Medical treatment may involve the use of intralesional corticosteroids, etanercept, tacrolimus, or oral minocycline. The definitive treatment is surgical excision, which can eliminate symptoms, prevent future complications, and prevent future interference with cancer screening.

## Conclusions

This case demonstrated diagnostic challenges of siliconomas. Siliconomas in the axillary, supraclavicular, and internal mammillary lymph nodes are well-known complications of silicone breast injection but can be mistaken for metastatic lymphadenopathy. Radiological studies are an invaluable tool for the accurate diagnosis of suspected siliconoma. All in all, this report highlights that the standard approach to diagnosis and management of siliconomas in an HIV patient is well tolerated and a viable course of action.
